# Accelerated Emergence of Self-Driving Laboratories for Accelerating Materials Discovery

**DOI:** 10.1021/acscentsci.5c01624

**Published:** 2026-02-18

**Authors:** Amanda K. Brown, Abhishek Soni, Daniel Lin, Curtis P. Berlinguette

**Affiliations:** † Department of Chemistry, 8166 The University of British Columbia, 2036 Main Mall, Vancouver, British Columbia V6T 1Z1, Canada; ‡ Department of Chemical and Biological Engineering, The University of British Columbia, 2360 East Mall, Vancouver, British Columbia V6T 1Z3, Canada; § Stewart Blusson Quantum Matter Institute, The University of British Columbia, 2355 East Mall, Vancouver, British Columbia V6T 1Z4, Canada; ∥ Canadian Institute for Advanced Research (CIFAR), 661 University Avenue, Toronto, Ontario M5G 1M1, Canada

## Abstract

The rise of self-driving laboratories (SDLs) marks a transformative era in materials science.

By leveraging automation and machine learning, SDLs provide unprecedented opportunities to rapidly explore complex chemical landscapes and accelerate discoveries.

In this Outlook, we explore the growth of the global SDL ecosystem in materials science, describe recent advances, and discuss how SDLs are poised to drive more discoveries and accelerate commercialization.

## Graphical abstract

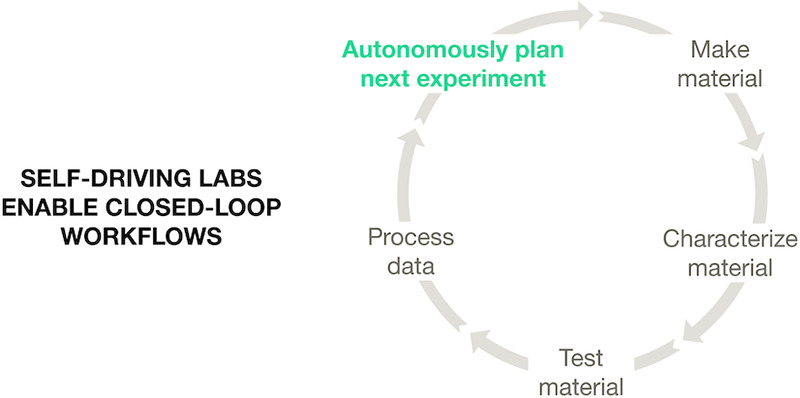

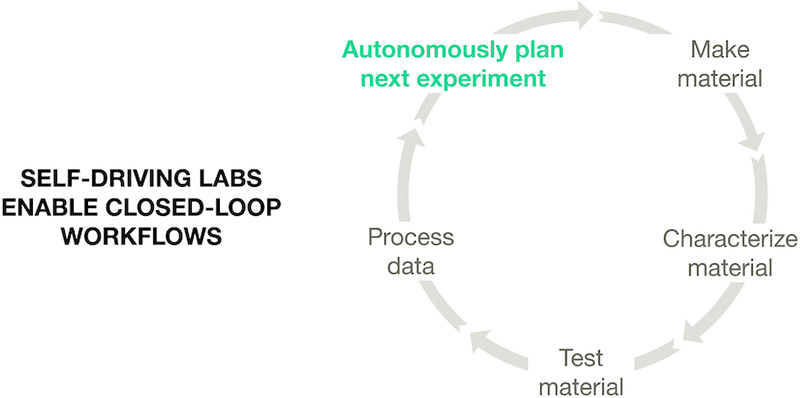

## Motivation

Materials discovery is a key element of the innovation cycle for developing new technologies. However, discovering effective materials among a vast array of conceivable formulations can take decades using conventional approaches. There are many tools (e.g., high-throughput experimentation) available to make millions of different materials, but the act of finding a material that is actually effective is challenging. SDLs aim to alleviate this materials discovery bottleneck by combining materials science with automation and machine learning. SDLs are designed to run an experiment, evaluate the result, and utilize this information to plan and execute the next experiment. A machine learning algorithm guides each automated round to maximize efficiency. Some SDL workflows may be wholly autonomous, while others may have steps that require human intervention.

## Origins of Self-Driving Laboratories (SDLs) for Materials Science

The roots of SDLs trace to mid-20th-century laboratory automation, such as Merrifield and Stewart’s automated solid-phase peptide synthesizer, which streamlined some routine experiments.^1^ Closed-loop SDL workflows (combining automated and autonomous experiment planning, execution, and analysis) emerged later, marked by milestones such as Adam[Bibr ref2] (2009) and Eve[Bibr ref3] (2015), which demonstrated autonomous workflows in genomics and drug discovery.

Automation has been more widely used in biology and chemistry (e.g., cell cultures, drug formulations, sample preparations, titrations, flow chemistries) relative to materials science. The slower progress in materials science and engineering stems in part from challenges associated with the physical and chemical diversity of materials systems, which often involve solids, powders, viscous slurries, or multiphase mixtures that are more difficult to manipulate robotically. In addition, many materials characterization techniques are also challenging to automate or are time-intensive and thus less amenable to accelerated workflows. These features, combined with sensitivities to environmental conditions such as temperature, humidity, or oxygen exposure, make the development of SDL-driven materials science workflows technically challenging.

In January 2018, Mission Innovation published an expert report calling for the development of SDLs for materials science that tackle these challenges.^4^ In response, the Canadian government supported us and collaborators in June 2018 to launch Project Ada, named in honor of Ada Lovelace, through which we built and demonstrated an SDL for thin-film materials research that is automated, autonomous, and adaptable for a variety of materials systems.^5–8^ Our program has since published two review articles. The first advocated for, and described the use of, “flexible automation” for SDL development in the context of materials science (with *flexible automation* defined as “the concept of using robotics to create reconfigurable automated experiments”).^9^ The second described characteristics of, and provided strategies for developing, effective SDLs.^10^ In this Outlook, we provide a contemporary view of how the global SDL ecosystem in material science is positioned to drive more discoveries and accelerate commercialization.

## Summary of Current Landscape and SDL-Driven Materials Exploration

The materials science SDL ecosystem has grown substantially over the past decade (Table 1).

**Table 1:** Examples of SDLs Reported for Applications Related to Materials Science

SDL	Corresponding author(s)	Application(s) and publication year(s)	Examples of hardware and software
ARES	Maruyama	Carbon nanotubes [Bibr ref11]−[Bibr ref12] [Bibr ref13] (2016; 2020; 2025)	Automated CVD chamber, Raman spectroscopy; AI-planner (random forest model; Bayesian optimization)
Ada	Berlinguette, Hein, Aspuru-Guzik	Hole-transport materials for perovskite solar cells[Bibr ref5] (2020)	Automated spin-coating, spray-coating, X-ray fluorescence, conductivity probe, imaging, CO_2_/bicarbonate electrolysis test cell; Bayesian optimization, ChemOS
		Adhesives[Bibr ref14] (2023)	
		Palladium thin films [Bibr ref6],[Bibr ref7] (2022; 2023)	
Mobile robotic chemist	Cooper	Photocatalysis [Bibr ref15],[Bibr ref16] (2020 and 2024)	Mobile robotic platform working in a conventional laboratory; Bayesian optimization
Inorganic thin-film SDL	Hitosugi, Shimizu, Ando	Inorganic thin films[Bibr ref17] (2020)	Automated radio frequency magnetron sputtering, conductivity probe; Bayesian optimization
BEAR	Brown, Morgan, Reyes	Additive manufacturing[Bibr ref18] (2020)	Automated five dual-extruder fused-deposition-modeling printers, scale, universal testing machine; Bayesian optimization
Otto/Clio	Whitacre, Viswanathan	Liquid battery electrolytes - aqueous[Bibr ref19] (2020) and nonaqueous[Bibr ref20] (2022)	Otto: Automated solution mixing, pH measurements, ionic conductivity measurements, electrochemical stability measurements; Dragonfly (a Bayesian optimization software package)
			Clio: Automated liquid handling, ionic conductivity, viscosity, density measurements; Dragonfly (a Bayesian optimization software package)
AMANDA Platform LineOne/SPINBOT	Brabec, Hauch, Wagner, Zhang	Organic photovoltaics[Bibr ref21] (2021)	AMANDA’s LineOne: Automated liquid handling, spin-coating, thermal treatment, imaging, UV–vis absorption, electrode evaporation, current-density-versus-voltage measurements; Gaussian process regression model
		Perovskite photovoltaics[Bibr ref22] (2023)	SPINBOT: Automated precipitation of microcrystalline lead-free perovskites, purification, drop-casting of film arrays, XRD, SEM, absorption, PL, and Raman spectroscopy; Bayesian optimization
A-Lab	Ceder, Zeng	Solid-state inorganic materials synthesis[Bibr ref23] (2023)	Automated powder dosing, sample heating, and X-ray diffraction; AlabOS;^24^ probabilistic ML models, active learning algorithm “ARROWS^3^”,^25^ LLMs for synthesis prediction
MicroFactory	Vak	Optimization of roll-to-roll printed photovoltaics[Bibr ref26] (2024)	Automated roll-to-roll fabricator (two slot-die deposition heads, slot-die head heaters, annealing systems), characterizer; artificial neural network
Polybot	Chan, Xu	Electronic polymers[Bibr ref27] (2025)	Automated modules for formulating, spin-coating, processing, processability testing, functionality testing; Bayesian optimization

Below we highlight five examples of SDLs that illustrate distinct materials science workflows across a range of materials classes, synthesis methods, and application domains.

Maruyama and colleagues first reported ARES in 2016.^11^ This SDL combines an automated chemical vapor deposition system with *in situ* characterization to explore growth parameters (temperature, pressure, and gas composition) for single-walled carbon nanotubes. ARES accelerated throughput by up to 100× relative to conventional experimentation. Over a series of >600 experiments, this SDL used an AI planner (a random forest model coupled with a genetic algorithm) to learn to synthesize nanotubes at targeted rates. They later used Bayesian optimization to improve growth rate by up to 8× relative to seed experiments,^13^ and reported the first multiobjective optimization campaign for single-walled carbon nanotube synthesis in which ARES maximized both overall yield and diameter control.^12^

The Berlinguette Group, in collaboration with the Hein and Aspuru-Guzik Groups, reported Ada in 2020, which was the first SDL for clean energy materials.^5^ Ada optimized optical and electronic properties of hole-transport materials used in perovskite solar cells by exploring film composition and processing conditions. We later used flexible automation to adapt Ada for “AdaCarbon” workflows, which explore materials and operating parameters for CO_2_ and reactive carbon electrolyzers. We demonstrated a 3× automated workflow acceleration relative to manual methods for electrode fabrication, characterization, and testing.^8^ We also used AdaCarbon to explore a 6-variable parameter space in fewer than 40 experiments to improve reactor yield, defined as the product of CO_2_ utilization and product formation rates.^28^ This work demonstrates how flexible automation enables a single SDL to be repurposed across multiple clean energy applications.

The Cooper Group reported an SDL (“mobile robotic chemist”) to optimize photocatalyst efficiency for hydrogen evolution in 2020.^15^ This SDL employs a mobile, free-roaming strategy, in which a robot moves between different instruments, performing liquid handling, in-line analysis, and sample processing. The mobile robotic chemist explored a 10-dimensional parameter space over 8 days by performing 688 experiments using a batched Bayesian search algorithm. The platform identified photocatalysts 6× as active compared to initial formulations. In 2024, the Cooper Group extended this approach to a workflow in which two autonomous mobile robots worked collaboratively, demonstrating how mobile robotic platforms can enable automation in conventional laboratories.^16^

In 2021, Brabec and colleagues reported AMANDA: an Autonomous Materials and Device Application Platform consisting of multiple SDLs.^21,29^ For example, their “LineOne (L1)” SDL produces and characterizes solution-processed thin-film devices, with an initial focus on organic photovoltaics and an estimated throughput of 216 devices per workday (which could be increased through parallel processed batches and continuous operation).^29^ They later shifted focus to perovskite materials and devices; their SPINBOT SDL explored 10 parameters related to preparing perovskite devices (in ambient air), and achieved efficiencies >23%.^22^ Work from Brabec and colleagues demonstrates how SDLs can accelerate both materials exploration and device performance optimization.

Ceder, Zeng, and colleagues reported A-Lab, an SDL for solid-state synthesis of inorganic materials, in 2023.^23^ A-Lab integrates computational phase-stability predictions, literature-derived synthesis knowledge, and active-learning algorithms to design and interpret experiments executed by a robotic workflow. Over 17 days of continuous operation, A-Lab attempted 58 oxide and phosphate targets and successfully realized 41 new compounds, guided by natural-language models. Analysis of unsuccessful syntheses provided mechanistic insight into synthesis limitations and yielded practical guidance for improving computational screening pipelines. This work illustrates the potential of combining computation with SDL workflows to accelerate the experimental realization of predicted materials.

Beyond these highlighted examples, the SDL-for-materials community is leveraging automation and machine learning across diverse materials systems and applications. Collectively, this community is accelerating experimentation and exploration of complex parameter spaces that would be slow or impractical to survey with conventional manual workflows.

## Challenges Associated with Materials Science SDLs

We highlight here common challenges that arise when designing, building, and using SDLs.

### Workflow Design

Arguably the most significant challenge in designing an SDL is defining an appropriate research problem to solve. The chosen problem must be valuable, and the problem must be addressable with an automated workflow with fast learning cycles. If the problem is not of genuine value to the practitioner’s community, then the SDL is not valuable. If any single step in the SDL workflow is too long, the acceleration factor will be low and the benefits of closed-loop optimization are lost.

Looking forward, SDL projects should prioritize the proper problems to solve, and workflows that are designed for speed. We will not be prescriptive here about which problems should be tackled; that decision is best left to the creative practitioner. In order to achieve speed, we do encourage the practitioner to incorporate creative strategies such as proxy objectives^5,30,31^ (faster measurements that correlate with the true objective) or multifidelity optimization^32–34^ (the strategic integration of faster, lower-accuracy measurements with slower, higher-accuracy measurements into the workflow). These strategies are not widely used.

### Workflow Flexibility

SDLs also need to be flexible. Workflows built around a narrowly defined objective or fixed sequence of operations can quickly and unexpectedly become obsolete. Practitioners should enter into the design of an SDL with the understanding that research priorities and experimental goals will almost certainly change. Plan for this evolution in advance.

A research group will invest years and significant resources into building or procuring an SDL. During this time the literature will continue to grow, the research team will change in composition, and researchers’ interests will evolve – thus, the original workflow may no longer be compatible with the lab’s needs. Flexible automation mitigates this risk: modular components can be isolated, swapped, or reconfigured, enabling individual elements to be repaired or adjusted without interrupting the entire SDL.^9^ This adaptability supports both long-term relevance and efficient troubleshooting when modules fail.

### Workflow Orchestration

Have you gone years without updating your phone? Unlikely. The same logic applies to a SDL. SDL software will evolve. Orchestration layers will change. Managing these updates requires real resources. Plan for them.

Do not assume seamless integration of new instruments. Many provide limited application programming interfaces (APIs), depend on proprietary control software, or expose only restricted low-level access. These constraints are structural, not edge cases, and they must be anticipated in SDL design.

The good news is that this situation is improving. As the SDL community matures, instrument manufacturers are increasingly willing to provide automation-ready interfaces or the access required to build custom control layers. In parallel, research groups have begun developing their own orchestration software, including ChemOS,^35^ Ares OS,^11,36^ and AlabOS.^24^ Extending any orchestration stack to a new SDL still requires nontrivial effort, but open sourcing enables reuse of core components and experiment design patterns, reducing the need to rebuild from first principles.^34^

### Data Management

Invest early and strategically into data management. Effective data management lays the groundwork for AI-driven experiment planning, where algorithms can leverage rich, structured data sets to make informed decisions and optimize workflows.

Design your SDL to automatically log every experimental parameter, track module performance, video-record each step, and maintain a machine-readable record of every experiment. The SDL should be capable of producing a lab notebook more complete and precise than any human could reasonably produce. A researcher’s ability to harness this wealth of information requires robust software infrastructure.

The integration, organization, and effective visualization of such data can be complex. Our group has found interactive dashboards valuable for data exploration and visualization.^37^

### Experimental Planning

AI-driven experiment planning also introduces challenges. For example, planning algorithms must make decisions despite experimental noise, which can arise from small differences in sample preparation, instrument sensitivity, environmental conditions, or other factors. Humans can often intuitively recognize and compensate for noise and outliers, but AI planners must quantify uncertainty. Approaches to address these challenges can include the use of surrogate models (computational models that approximate experimental outcomes and reduce the number of physical experiments needed to effectively explore a chosen parameter space) to guide predictions and account for experimental noise, as well as interpretable AI models that allow humans to validate or adjust experiment proposals. Previous reviews, perspectives, and articles provide more detailed discussions of SDL experiment planning, including challenges, trends, and opportunities.^34,38–42^

## The Future of SDLs Will Result in More Discoveries

### SDLs Will Be More Flexible

The establishment of a research group’s first functional SDL is a substantial undertaking, requiring the accumulation of internal expertise or collaborators with expertise in automation, experimental design, algorithmic planning, and data management, as well as careful selection of scientific problems amenable to SDL exploration. Once this foundational capacity is in place, SDLs that leverage flexible automation can be adapted with relative ease (compared to the time investment needed to establish an inaugural SDL workflow) to diverse materials systems, enabling rapid iteration and accelerated exploration of complex parameter spaces. As a community cohort transitions from a largely “platform development” phase to an iterative design-upgrade-leverage phase, we expect to see a growing body of discoveries that can be directly attributed to SDL-driven investigation.

### SDLs Will Be More Accessible

Various tools and synergistic technologies are lowering the technical barriers and costs associated with building and operating SDLs.

On the hardware side, we have previously described how collaborative robots with built-in safety limits, rapid-prototyping tools such as 3D printers and laser cutters, and inexpensive digital cameras have made SDL development more affordable.^9^ Open-access hardware designs and standardized protocols can further streamline platform construction. For example, the “Frugal Twin” concept proposes building simplified, low-cost SDLs (<$50,000) as test beds by using off-the-shelf components to reduce hardware costs and design time.^43^

On the software side, AI-based tools such as ZoMBI^44^ and multifidelity ML models^45^ have the potential to accelerate experiment planning and data analysis by predicting promising experimental conditions, prioritizing high-value measurements, and helping researchers explore the trade-offs that arise during decision-making. Large language models (LLMs) can increase accessibility by generating experimental code, managing data workflows, and interfacing with laboratory hardware from natural-language instructions, enabling practitioners with limited programming experience to operate SDLs. Beyond accessibility, LLMs may also support substantially more complex experimental workflows, reasoning, and tool integration.

Together, hardware and software developments are increasing the accessibility of SDLs and broadening participation across the scientific community. As more SDLs are built and operated, addressing data standardization will become increasingly important to ensure that experimental data, protocols, and workflows can be shared and used reliably across laboratories. A detailed discussion of standardization is beyond the scope of this Outlook; however, collaborative initiatives such as the ones discussed below are poised to lead the charge.

### SDLs Will Be More Collaborative

A number of initiatives have been established to build SDLs, educate a new generation of researchers, and foster collaboration between government, academia, and industry. For example, the Acceleration Consortium is “a global network of government, academia, and industry working to accelerate the discovery of materials”^46^ that was awarded a $200 M grant from the Canada First Research Excellence Fund (the largest government grant awarded to a university in Canadian history to date).^47^ The Canadian government has funded additional initiatives involving SDL development, including the National Research Council of Canada’s $57 M Materials for Clean Fuels Challenge Program^48^ and the Advanced Materials Research Facility in Mississauga, Ontario.^49^ Canada and Germany are partners in the German-Canadian Materials Acceleration Center (GC-MAC) focused on accelerating discovery, demonstration, and deployment of materials for sustainable energy technologies.^50^ Outside of Canada, the European Commission funded the 20 M Euro Battery Interface Genome – Materials Acceleration Platform (BIG-MAP) project through the Battery 2030+ initiative,^51^ and Argonne National Laboratory in the United States has launched multiple SDL projects.^52^

These coordinated efforts can help address one of the central challenges facing the SDL community: access to platforms, data, and expertise. By establishing shared infrastructure, open-access data frameworks, and cross-institutional training programs, these initiatives lower the barrier to entry for groups without the resources to build their own SDL in-house from scratch. In doing so, these collaborative endeavors can help support a more democratized and globally distributed SDL ecosystem in which discoveries can increasingly arise from collaborative networks rather than isolated laboratories.

### SDLs Are Set to Accelerate Commercialization

We are seeing trends in manufacturing and scalability considerations factored into earlier stage development.^53^ In our lab, we transitioned our SDL from automated spin-coating to automated spray-coating as a more scalable deposition method.^7^ As SDL capabilities in materials science mature, industry partners are uniquely positioned to scale these platforms, integrate them into industrial R&D and manufacturing workflows, and translate discovery into commercial products. The last several years have seen the emergence of a rapidly expanding commercial ecosystem built around SDL concepts, with multiple venture backed companies developing autonomous laboratory platforms, cloud-based experimentation services, and AI-driven optimization engines. These efforts collectively represent hundreds of millions of dollars of private investment flowing into automated science, signaling that SDLs are moving beyond bespoke academic prototypes into scalable technologies with industrial relevance. Industry participation brings application driven problem statements, robust engineering expertise, and resources for large scale data generation and infrastructure, while SDL-enabled collaboration models allow companies to benefit from emerging automation technologies and cutting-edge academic advances. As academic, industrial, and government efforts continue to converge, the SDL ecosystem will be increasingly poised to accelerate the discovery of new materials and translate SDL driven breakthroughs into deployable technologies that address real world needs.

### Future Laboratories with SDLs Are Coming Fast

Taken together, the advances described above signal a pivotal moment for materials science.

SDLs are evolving from bespoke research tools into widely accessible, flexible, and collaborative engines that replace lengthy trial-and-error cycles and accelerate the pace of discovery.

Government investment, academic innovation, and growing industrial participation are converging to create an ecosystem where data, automation, and intelligent experimentation reinforce one another.

As barriers to adoption fall and applications broaden, SDLs are poised to reshape how materials are discovered, developed, and deployed.

The result will be faster innovation cycles, more efficient use of resources, and a clearer pathway from fundamental research to commercial technologies that meet urgent global challenges.

## Supplementary Material



## Notes

The Supporting Information file contains a supplementary figure (map showing self-driving laboratories for materials science), a list of institutions included in the map, and associated references. (PDF)
