# Supplementary material for: Accelerated Emergence of Self-Driving Laboratories for Accelerating Materials Discovery
Source: ACS Cent Sci. 2026 Feb 18;12(3):300–6. doi: 10.1021/acscentsci.5c01624 (PMC13107214; doi:10.1021/acscentsci.5c01624)
Supplement: Supplementary file 1 [file oc5c01624_si_001.pdf]

Supporting Information

Accelerated Emergence of Self-Driving Laboratories for Accelerating Materials Discovery

Amanda K. Brown,[1] Abhishek Soni,[1] Daniel Lin,[1] Curtis P. Berlinguette*[1,2,3,4]

[1]Department of Chemistry, The University of British Columbia, 2036 Main Mall, Vancouver, British Columbia, V6T 1Z1, Canada.

[2]Department of Chemical and Biological Engineering, The University of British Columbia, 2360 East Mall, Vancouver, British Columbia, V6T 1Z3, Canada.

[3]Stewart Blusson Quantum Matter Institute, The University of British Columbia, 2355 East Mall, Vancouver, British Columbia, V6T 1Z4, Canada.

[4]Canadian Institute for Advanced Research (CIFAR), 661 University Avenue, Toronto, Ontario, M5G 1M1, Canada.

*Corresponding author: Curtis P. Berlinguette (cberling@chem.ubc.ca)

Supplementary figure

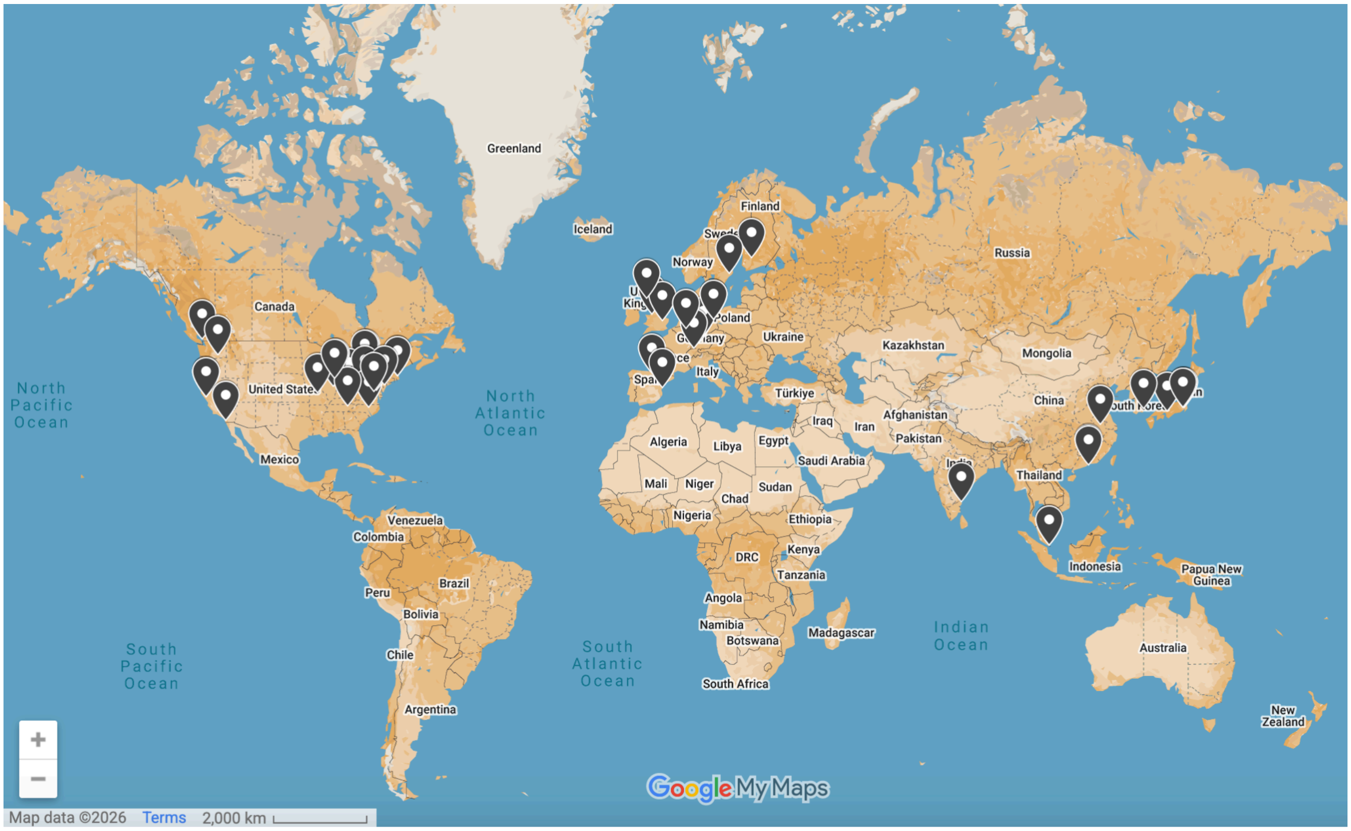

Supplementary figure 1. Map showing self-driving laboratories for materials science (created using Google Maps. Map data ©2026).

Institutions included in Supplementary figure 1

- University of British Columbia[1–4]
- University of California, Berkeley[5]
- Lawrence Berkeley National Laboratory[6]
- Pacific Northwest National Laboratory[7]
- University of California, San Diego[8]
- University of Missouri[9]
- Argonne National Laboratory[10]
- University of Illinois at Urbana-Champaign[11]
- University of Chicago[12]
- Oak Ridge National Laboratory[13]
- Air Force Research Laboratory[14,15]
- National Research Council of Canada[16]
- The University of Toronto[17–19]
- North Carolina State University[20–23]
- National Institute of Standards and Technology[24]

- Carnegie Mellon University[25]
- Johns Hopkins University[26]
- The State University of New Jersey[27,28]
- Massachusetts Institute of Technology[29]
- Boston University[30]
- Centre for Cooperative Research on Alternative Energies (CIC energiGUNE)[31,32]
- University of Glasgow[33]
- University of Liverpool[34,35]
- Universitat Jaume[36]
- University of Cambridge[37]
- Forschungszentrum Jülich GmbH[38–40]
- Swiss Federal Laboratories for Materials Science and Technology[41]
- Helmholtz Institute Ulm[42,43]
- Helmholtz-Institute Erlangen-Nürnberg[44]
- Federal Institute for Materials Research and Testing (BAM)[45]
- Dunia Innovations[46]
- VTT Technical Research Centre of Finland Ltd[47]
- KTH Royal Institute of Technology[48]
- Nanyang Technological University[49]
- National University of Singapore[50]
- Indian Institute of Technology Madras[51]
- Chinese Academy of Sciences[52]
- University of Science and Technology of China[53]
- Institute for Basic Science[54]
- Osaka University[55]
- Tokyo Institute of Technology[56]
- University of Tokyo[57,58]
- Institute of Science Tokyo[59]
- The Chinese University of Hong Kong[60]

Note: For instances where a publication involved collaboration amongst institutions, the first institution listed for the first author was mapped.

References

1. MacLeod, B. P. *et al.* Self-driving laboratory for accelerated discovery of thin-film materials. *Sci. Adv.* **6**, eaaz8867 (2020).
2. MacLeod, B. P. *et al.* A self-driving laboratory advances the Pareto front for material properties. *Nat. Commun.* **13**, 995 (2022).
3. Rupnow, C. C. *et al.* A self-driving laboratory optimizes a scalable process for making functional coatings. *Cell Rep. Phys. Sci.* **4**, 101411 (2023).
4. Rooney, M. B. *et al.* A self-driving laboratory designed to accelerate the discovery of adhesive materials. *Digit. Discov.* **1**, 382–389 (2022).
5. Szymanski, N. J. *et al.* An autonomous laboratory for the accelerated synthesis of novel materials. *Nature* **624**, 86–91 (2023).
6. Halder, A. *et al.* AI‐driven robot enables synthesis‐property relation prediction for metal Halide perovskites in humid atmosphere. *Adv. Energy Mater.* **15**(34), 2502294 (2025).
7. Noh, J. *et al.* An integrated high-throughput robotic platform and active learning approach for accelerated discovery of optimal electrolyte formulations. *Nat. Commun.* **15**, 2757 (2024).
8. Cakan, D. N. *et al.* PASCAL: the perovskite automated spin coat assembly line accelerates composition screening in triple-halide perovskite alloys. *Digit. Discov.* **3**, 1236–1246 (2024).
9. Xie, Y. *et al.* Accelerate synthesis of metal-organic frameworks by a robotic platform and Bayesian optimization. *ACS Appl. Mater. Interfaces* **13**, 53485–53491 (2021).
10. Wang, C. *et al.* Autonomous platform for solution processing of electronic polymers. *Nat. Commun.* **16**, 1498 (2025).
11. Oh, I. *et al.* The Electrolab: An open-source, modular platform for automated characterization of redox-active electrolytes. *Device* **1**, 100103 (2023).
12. Zheng, Y. B. *et al.* A self-driving physical vapor deposition system making sample-specific decisions on the fly. *Npj Comput. Mater.* **11**, 327 (2025).
13. Vasudevan, R. K. *et al.* Autonomous experiments in scanning probe microscopy and spectroscopy: Choosing where to explore polarization dynamics in ferroelectrics. *ACS Nano* **15**, 11253–11262 (2021).
14. Nikolaev, P. *et al.* Autonomy in materials research: a case study in carbon nanotube growth. *Npj Comput. Mater.* **2**, 16031 (2016).
15. Waelder, R., Kim, W., Pitt, M. A., Myung, J. I. & Maruyama, B. Multi-objective Bayesian optimization of carbon nanotube yield and diameter control at synthesis. *APL Mach. Learn.* **3**, 026114 (2025).
16. Fatehi, E., Thadani, M., Birsan, G. & Black, R. W. A critical evaluation of a self-driving laboratory for the optimization of electrodeposited earth-abundant mixed-metal oxide catalysts for the oxygen evolution reaction (OER). *arXiv [physics.app-ph]* (2023). doi:10.48550/arXiv.2305.12541.
17. Park, H. S. *et al.* A self-driving lab for discovering tunable and soluble organic lasers. *ChemRxiv* (2025) doi:10.26434/chemrxiv-2025-0vr9f-v2.
18. Wu, T. C. *et al.* A materials acceleration platform for organic laser discovery. *Adv. Mater.* **35**, 2207070 (2023).
19. Strieth-Kalthoff, F. *et al.* Delocalized, asynchronous, closed-loop discovery of organic laser

emitters. *Science* **384**, eadk9227 (2024).

20. Delgado-Licona, F. *et al.* Flow-driven data intensification to accelerate autonomous inorganic materials discovery. *Nat. Chem. Eng.* **2**, 436–446 (2025).
21. Wang, T. *et al.* Sustainable materials acceleration platform reveals stable and efficient wide-bandgap metal halide perovskite alloys. *Matter* **6**, 2963–2986 (2023).
22. Sadeghi, S. *et al.* A self-driving fluidic lab for data-driven synthesis of lead-free perovskite nanocrystals. *Digit. Discov.* **4**, 1722–1733 (2025).
23. Xu, J. *et al.* Autonomous multi-robot synthesis and optimization of metal halide perovskite nanocrystals. *Nat. Commun.* **16**, 7841 (2025).
24. Beaucage, P. A. & Martin, T. B. The autonomous formulation laboratory: An open liquid handling platform for formulation discovery using X-ray and neutron scattering. *Chem. Mater.* **35**, 846–852 (2023).
25. Dave, A. *et al.* Autonomous discovery of battery electrolytes with robotic experimentation and machine learning. *Cell Rep. Phys. Sci.* **1**, 100264 (2020).
26. Lin, D.-Z. *et al.* A high-throughput experimentation platform for data-driven discovery in electrochemistry. *Sci. Adv.* **11**, eadu4391 (2025).
27. Lee, J. *et al.* A fully automated platform for photoinitiated RAFT polymerization. *Digit. Discov.* **2**, 219–233 (2023).
28. Tamasi, M. J. & Gormley, A. J. Biologic formulation in a self-driving biomaterials lab. *Cell Rep. Phys. Sci.* **3**, 101041 (2022).
29. Zhang, Z. *et al.* A multimodal robotic platform for multi-element electrocatalyst discovery. *Nature* **647**, 390–396 (2025).
30. Gongora, A. E. *et al.* A Bayesian experimental autonomous researcher for mechanical design. *Sci. Adv.* **6**, eaaz1708 (2020).
31. Monterrubio, I. *et al.* Affordable automated modules for lab-scale high-throughput synthesis of inorganic materials. *Chemistry* **31**, e02072 (2025).
32. Ismail, M. *et al.* ADEL: an automated drop-cast electrode setup for high-throughput screening of battery materials. *Digit. Discov.* **4**, 943–953 (2025).
33. Laws, K. *et al.* An autonomous electrochemical discovery robot that utilises probabilistic algorithms: Probing the redox behaviour of inorganic materials. *ChemElectroChem* **11**, (2024).
34. Burger, B. *et al.* A mobile robotic chemist. *Nature* **583**, 237–241 (2020).
35. Dai, T. *et al.* Autonomous mobile robots for exploratory synthetic chemistry. *Nature* **635**, 890–897 (2024).
36. Tinajero, C., Zanatta, M., Sánchez-Velandia, J. E., García-Verdugo, E. & Sans, V. Reac-Discovery: an artificial intelligence-driven platform for continuous-flow catalytic reactor discovery and optimization. *Nat. Commun.* **16**, 9062 (2025).
37. Pomberger, A. *et al.* Automated pH adjustment driven by robotic workflows and active machine learning. *Chem. Eng. J.* **451**, 139099 (2023).
38. Zhang, J., Hauch, J. A. & Brabec, C. J. Toward self-driven autonomous material and device acceleration platforms (AMADAP) for emerging photovoltaics technologies. *Acc. Chem. Res.* **57**, 1434–1445 (2024).
39. Zhang, J. *et al.* Optimizing perovskite thin‐film parameter spaces with machine learning‐guided robotic platform for high‐performance perovskite solar cells. *Adv. Energy Mater.* **13**, 2302594

(2023).
40. Du, X. *et al.* Elucidating the full potential of OPV materials utilizing a high-throughput robot-based platform and machine learning. *Joule* **5**, 495–506 (2021).
41. Senocrate, A. *et al.* Parallel experiments in electrochemical CO_2 reduction enabled by standardized analytics. *Nat. Catal.* **7**, 742–752 (2024).
42. Rahmanian, F., Fuchs, S., Zhang, B., Fichtner, M. & Stein, H. S. Autonomous millimeter scale high throughput battery research system. *Digit. Discov.* **3**, 883–895 (2024).
43. Vogler, M. *et al.* Autonomous battery optimization by deploying distributed experiments and simulations. *Adv. Energy Mater.* **14**, 2403263 (2024).
44. Zhang, J. *et al.* A fully robotic platform for optimizing the high-dimensional processing parameter space of perovskite thin-films. *SSRN Electron. J.* (2022). doi:10.2139/ssrn.4309089.
45. Zaki, M., Prinz, C. & Ruehle, B. A self-driving lab for nano- and advanced materials synthesis. *ACS Nano* **19**, 9029–9041 (2025).
46. Ng, M. T.-K., Ismail, A. S. M. & Hammer, A. J. S. A catalyst acceleration platform toward realizing the energy transition. *Matter* **5**, 4179–4186 (2022).
47. Antikainen, A. *et al.* SOLID-MAP: Development of a materials acceleration platform for high-entropy alloys. *J. Mater. Eng. Perform.* **34**, 30132–30137 (2025).
48. Lei, B., Svensson, P. H., Yushmanov, P. & Kloo, L. AURORA - an automatic robotic platform for materials discovery. *ACS Appl. Mater. Interfaces* **17**, 26701–26709 (2025).
49. Ng, L. W. T. *et al.* A printing-inspired digital twin for the self-driving, high-throughput, closed-loop optimization of roll-to-roll printed photovoltaics. *Cell Rep. Phys. Sci.* **5**, 102038 (2024).
50. Su, J. *et al.* Intelligent synthesis of magnetic nanographenes via chemist-intuited atomic robotic probe. *Nat. Synth.* **3**, 466–476 (2024).
51. Talluri, Y. N., Sankaranarayanan, S. K., Fry, H. C. & Batra, R. Discovery of unconventional and nonintuitive self-assembling peptide materials using experiment-driven machine learning. *Sci. Adv.* **11**, eadt9466 (2025).
52. Zhao, H. *et al.* A robotic platform for the synthesis of colloidal nanocrystals. *Nat. Synth.* **2**, 505–514 (2023).
53. Zhu, Q. *et al.* Automated synthesis of oxygen-producing catalysts from Martian meteorites by a robotic AI chemist. *Nat. Synth.* **3**, 319–328 (2023).
54. Jia, Y. *et al.* Robot-assisted mapping of chemical reaction hyperspaces and networks. *Nature* **645**, 922–931 (2025).
55. Yotsumoto, Y., Nakajima, Y., Takamoto, R., Takeichi, Y. & Ono, K. Autonomous robotic experimentation system for powder X-ray diffraction. *Digit. Discov.* **3**, 2523–2532 (2024).
56. Shimizu, R., Kobayashi, S., Watanabe, Y., Ando, Y. & Hitosugi, T. Autonomous materials synthesis by machine learning and robotics. *APL Mater.* **8**, 111110 (2020).
57. Nagai, K. *et al.* Sample-efficient parameter exploration of the powder film drying process using experiment-based Bayesian optimization. *Sci. Rep.* **12**, 1615 (2022).
58. Asano, Y., Okada, K., Nakagawa, S., Yoshie, N. & Shiomi, J. Automation of polymer pressing by robotic handling with in-process parameter optimization. *Rob. Auton. Syst.* **185**, 104868 (2025).
59. Nishio, K. *et al.* A digital laboratory with a modular measurement system and standardized data format. *Digit. Discov.* **4**, 1734–1742 (2025).
60. Li, J. *et al.* Autonomous discovery of optically active chiral inorganic perovskite nanocrystals

through an intelligent cloud lab. *Nat. Commun.* **11**, 2046 (2020).
